# Visfatin Affects the Transcriptome of Porcine Luteal Cells during Early Pregnancy

**DOI:** 10.3390/ijms25042339

**Published:** 2024-02-16

**Authors:** Grzegorz Kopij, Marta Kiezun, Kamil Dobrzyn, Ewa Zaobidna, Barbara Zarzecka, Agnieszka Rak, Tadeusz Kaminski, Barbara Kaminska, Nina Smolinska

**Affiliations:** 1 Faculty of Biology and Biotechnology, University of Warmia and Mazury in Olsztyn, Oczapowskiego 1A, 10-719 Olsztyn, Poland; grzegorz.kopij@uwm.edu.pl (G.K.); marta.kiezun@uwm.edu.pl (M.K.); kamil.dobrzyn@uwm.edu.pl (K.D.); ewa.zaobidna@uwm.edu.pl (E.Z.); barbara.zarzecka@uwm.edu.pl (B.Z.); tkam@uwm.edu.pl (T.K.); barbara.kaminska@uwm.edu.pl (B.K.); 2 Institute of Zoology and Biomedical Research, Jagiellonian University in Krakow, Gronostajowa 9, 30-387 Krakow, Poland; agnieszka.rak@uj.edu.pl

**Keywords:** adipokine, RNA-Seq, corpus luteum, alternative splicing, angiogenesis, differential expressed genes, signalling pathways

## Abstract

Visfatin/NAMPT (VIS), the hormone exerting a pleiotropic effect, is also perceived as an important factor in the regulation of reproductive processes and pregnancy maintenance. Previous studies confirmed its involvement in the control of porcine pituitary and ovary function. In this study, we hypothesized that VIS may affect the global transcriptome of luteal cells and thus regulate the functioning of the ovaries. Illumina’s NovaSeq 6000 RNA sequencing was performed to investigate the differentially expressed genes (DEGs) and long non-coding RNAs (DELs) as well as the occurrence of differential alternative splicing events (DASs) in the porcine luteal cells exposed to VIS (100 ng/mL) during the implantation period. The obtained results revealed 170 DEGs (99 up- and 71 downregulated) assigned to 45 functional annotations. Moreover, we revealed 40 DELs, of which 3 were known and 37 were described for the first time. We identified 169 DASs events. The obtained results confirmed a significant effect of VIS on the transcriptome and spliceosome of luteal cells, including the genes involved in the processes crucial for successful implantation and pregnancy maintenance as angiogenesis, steroidogenesis, inflammation, cell development, migration, and proliferation.

## 1. Introduction

The first month of gestation is crucial to reproductive success. On days 15–16, the pregnancy is either established successfully (implantation) or lost, i.a. as a result of insufficient interaction between the embryos and uterus. The ovaries are reproductive organs that directly mediate ovulation and have a profound impact on reproduction efficiency, but also on pregnancy establishment. The basic condition of a successful pregnancy and the highest ratio of embryo survival is the formation of ovarian luteal tissue. The corpus luteum (CL) plays a crucial role throughout gestation in many species of animals, and luteolysis at any stage of pregnancy will result in pregnancy loss. CL remains the main source of this hormone. Its development and maintenance during pregnancy depend on embryo–maternal crosstalk [1,2]. Pregnancy is tightly regulated by the internal environment, various endocrine factors, and the expression of a large number of genes. Gene expression studies within the porcine reproductive tissues are limited. Transcriptomic changes in the endometrial tissue have been analysed during pregnancy [3,4,5,6,7,8]. Scarce data from ovarian transcriptome analyses revealed differentially expressed genes (DEGs) with a few gene networks involved in steroidogenesis, apoptosis, prostaglandin synthesis, and angiogenesis [9,10,11]. A comprehensive understanding of the regulatory mechanism of pregnancy establishment and maintenance in domestic animals is important for manipulating animals’ productivity and resolving reproductive problems such as infertility.

Identified in 2004 by Fukuhara et al. [12], visfatin (VIS), also termed nicotinamide phosphoribosyltransferase (NAMPT) or pre-B cell colony-enhancing factor (PBEF), is a 52 kDa protein [13]. In humans, its active form contains two 491-amino-acid monomers. As for now, no VIS receptor has been identified, but it is suggested to be the tyrosine-phosphorylate insulin receptor in the hepatocytes, myocytes, and adipocytes [for review, see [14]]. VIS gene and protein sequences are presented as highly conservative in various animals [15]. Recently, VIS was also described as the adipokine mainly produced by the adipose tissue as well as tissues of the hypothalamic–pituitary–gonadal (HPG) axis in various species: mice, chicken, sheep, rats, pigs, and humans [16,17,18,19,20,21]. Mlyczynska et al. [22] indicated the differential pattern of VIS gene and protein expression in the porcine CL depending on the hormonal status, phase of the oestrous cycle, and stage of pregnancy. VIS is described as a novel protein mediator involved in metabolic and immune disorders (obesity, type II diabetes) [23,24,25], as well as the control of energy and glucose homeostasis, inflammation, lipid metabolism [26], cell differentiation [27], and angiogenesis [26,28,29]. Some data suggest that VIS, like other adipokines [17,19], could be involved in regulating female fertility. Administration of the adipokine during superovulation induces a developmental competency of oocytes [30]. VIS is expressed in the human foetal membranes and is present throughout gestation in the amniotic epithelium and mesenchymal cells [31]. In women, VIS increased fertility potential, implantation, and placentation [32,33]. In cattle, VIS takes part in ovarian steroidogenesis [19]. Our latest study indicated the modulatory effect of luteinizing hormone (LH) and insulin (INS) on the concentration of VIS protein in the porcine luteal cells (LCs) and their culture media depending on the phase of the oestrous cycle [22]. Moreover, we demonstrated that blocking the ERK1/2 kinase pathway abolishes the LH-, PGE_2_-, and progesterone (P_4_)-induced effects on VIS secretion by LCs [22]. It is also worth mentioning that VIS, through the MAPK and AMPK pathways, affects P_4_ synthesis in the porcine LCs [34]. Therefore, we suggest that VIS may regulate the transcriptome of porcine LCs.

The role of VIS in reproduction, fertility, and steroidogenesis processes is still undeniable. Most of these functions are controlled by LCs—the fundamental place for the production and secretion of steroids. However, despite VIS’s impact on reproductive processes, our knowledge of its impact on LCs is very limited. We hypothesized that VIS affects the transcriptome of the porcine LCs during early pregnancy, thereby influencing the expression of genes whose products control CL function. This is the first study that demonstrated the effect of VIS on DEGs, differentially expressed long noncoding RNAs (DELs), and differential alternative splicing events (DASs) occurring in LCs. Our discussion will be focused on the most interesting changes in the transcriptome and spliceosome that can be related to reproductive success and pregnancy.

## 2. Results

### 2.1. RNA Sequencing Data

The raw sequence data were submitted to the European Nucleotide Archive database (https://www.ebi.ac.uk/ena) with the accession number PRJEB61451 (accessed on 19 April 2023). The number of raw paired-end reads obtained by RNA-Seq was 579,987,746, with an average of 57.99 million reads per sample. The overall number of processed reads aligned to the *Sus scrofa* reference genome, ver. 96, was 474,172,478. On average, 87.83% of reads were uniquely mapped. The analysis affirmed that a mean of 43.81% of reads were mapped to coding DNA sequence regions (CDSs), while 19.38% aligned to untranslated regions (UTRs), 31.86% to introns, and 4.95% to intergenic regions. The above-mentioned sequencing data for individual samples are presented in Supplementary Table S1.

### 2.2. Analysis of Differentially Expressed Genes

**Figure 1:**
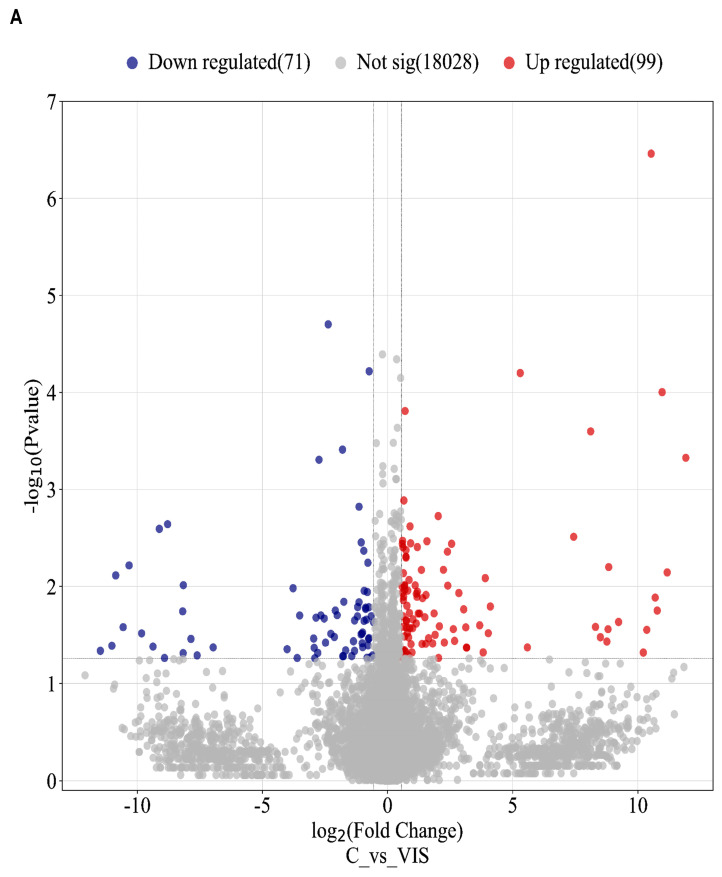

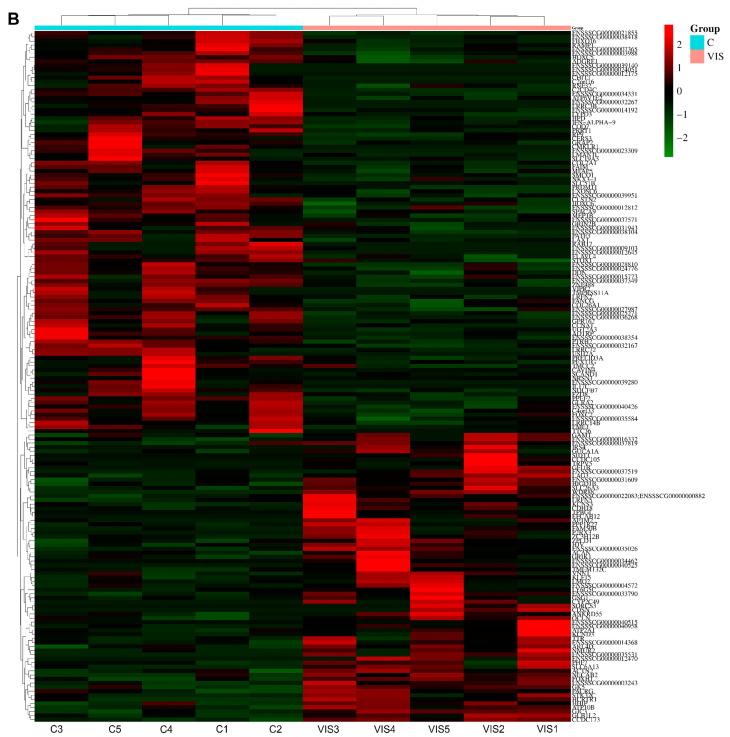
Visualization of statistically significant differentially expressed genes (DEGs) (*p* < 0.05) in response to visfatin (VIS). (**A**) The volcano plot showing the log_2_ of fold change (log_2_FC) plotted against −log_10_(*p*-value) for DEGs between the VIS-treated group and the control group. (**B**) The heatmap presenting expression profiles of DEGs. The data are displayed in a grid, where each row represents the gene and each column represents the sample of biological replicates of the control (C 1–5) or VIS-treated group (VIS 1–5). The colour and intensity of the boxes are used to represent the normalized changes (Z-score) in gene expression. Red boxes represent upregulated genes and green boxes represent downregulated genes. Abbreviations: C—control group; VIS—visfatin-treated group.

An expression profile analysis indicated the significant expression differences, based on absolute log_2_ of fold change (log_2_(FC) ≥ |0.56|; (*p*-value < 0.05) of 189 TARs within the VIS-treated group compared to the control group; among them, 170 DEGs were found. Of them, 99 were upregulated, while 71 were downregulated. The calculated log_2_FC values of the DEGs ranged from −11.48 (*ENSSSCG00000012470*) to 11.92 (*ENSSSCG00000039280*) (Supplementary Table S2). The expression profiles of all DEGs presented normalized counts and −log_10_(*p*-value) in the volcano plot (Figure 1A). The expression profile of all DEGs is shown in the heatmap (Figure 1B).

**Figure 2:**
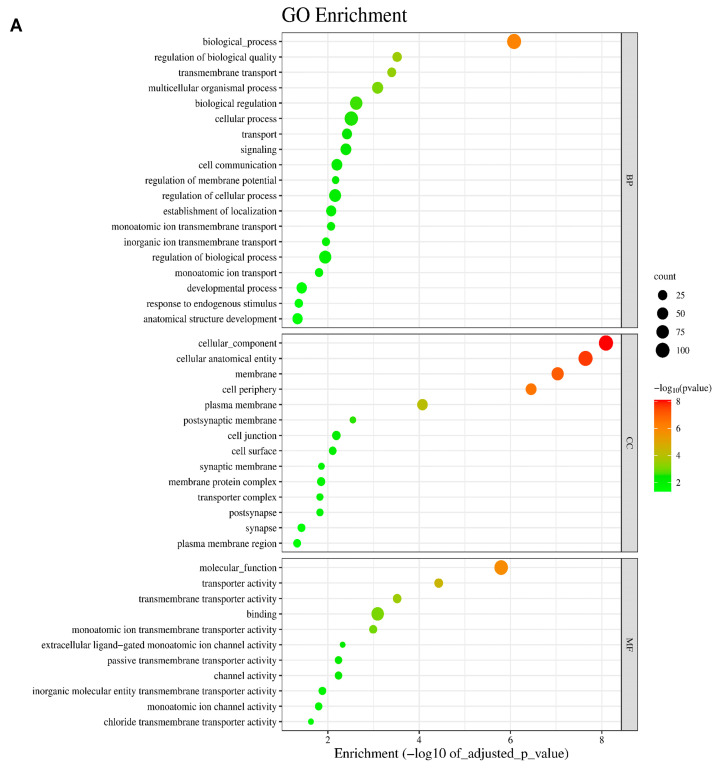

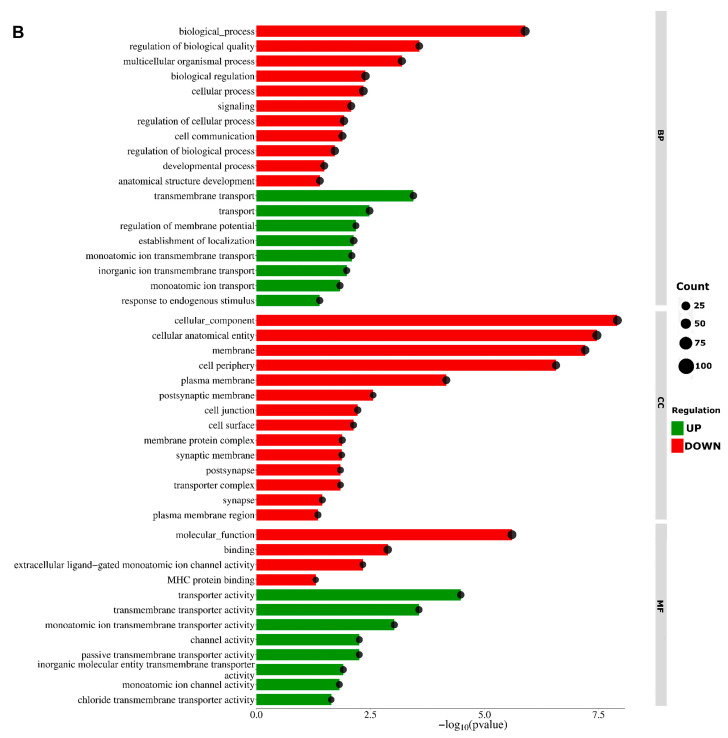
Summary of Gene Ontology (GO) term enrichment analysis with the differentially expressed genes (DEGs) evaluated in the luteal cells treated with visfatin (VIS). (**A**) Colour of dots represents statistically significant DEGs and their enrichment in the ontology terms by g:Profiler tool. The size of the dots represents the number of DEGs annotated to GO terms. (**B**) The colour of bars represents the ratio between up- and downregulated genes. Green bars show GO terms with the advantages of upregulated DEGs, whereas red bars represent terms that mainly consist of downregulated DEGs. GO enrichment terms are classified into the biological process (BP), cellular components (CC), and molecular function (MF). The Y-axis describes GO terms’ IDs (terms are presented in Supplementary Table S3) and the X-axis shows −log_10_(adjusted *p*-value) for each enriched GO term.

**Figure 3:**
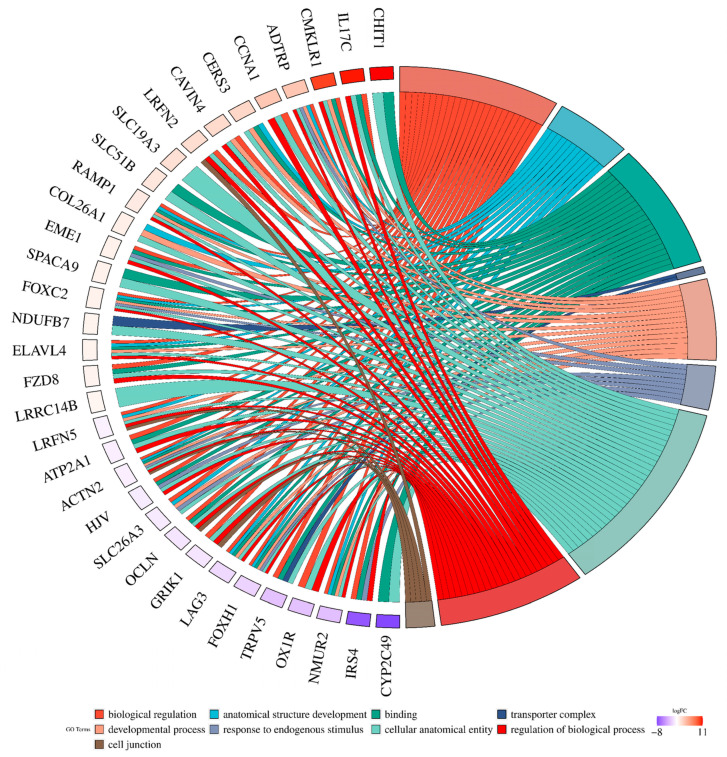
Visualisation of the chosen statistically significant DEGs and their enrichment in the ontology terms detected by g:Profiler tool. The circular plot shows the selected GO molecular functions (MF), biological process (BP), and cellular components (CC) enriched by DEGs evaluated in the luteal cells treated with VIS (*p* < 0.05, log_2_FC > |0.56|). Upregulated DEGs are displayed in red, whereas downregulated ones are displayed in blue. Chords connect protein names with biological process GO term groups. Each GO term is represented by one coloured line.

**Figure 4:**
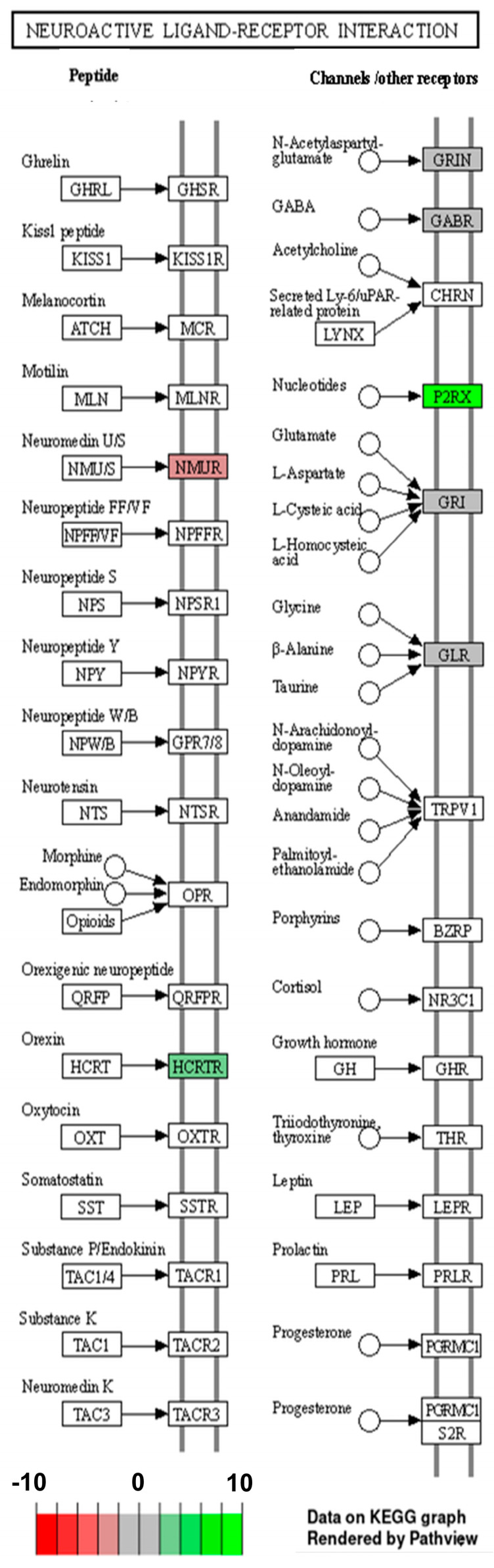
The neuroactive ligand–receptor interaction pathway is enriched by seven differentially expressed genes (DEGs). The red box shows upregulated genes and the green box represents downregulated genes estimated based on log_2_(FC). The scheme was shortened to describe enriched DEGs.

A GO analysis was performed for all 135 annotated DEGs under the influence of VIS. The genes were catalogized to 45 real-time terms (*p*-adjusted < 0.05): “cellular components” (CC)—14 terms; “molecular function” (MF)—12 terms; and “biological process” (BP)—19 terms (Figure 2A). From the CC category, the highest numbers of DEGs were annotated to the cellular component (GO:0005575; 110 DEGs), cellular anatomical entity (GO:0098590; 108 DEGs), membrane (GO:0016020; 68 DEGs), cellular periphery (GO0071944; 47 DEGs), and plasma membrane (GO:0005886; 40 DEGs), underscoring the diverse impact of VIS on cell organization. Within the MF category, DEGs were ascribed to transporter activity (GO:0005215; 19 DEGs), transmembrane transporter activity (GO:0022857; 17 DEGs), monoatomic ion transmembrane transporter activity (GO:0015075; 13 DEGs), and inorganic molecular entity transmembrane transporter activity (GO:0051032; 11 DEGs). These functions suggest that VIS may influence molecular processes within the cell. In the BP category, the significant GO terms with the most annotated DEGs were the cellular process (GO:0009987; 92 DEGs), biological regulation (GO:0065007; 69 DEGs), the regulation of biological processes (GO:0050789; 65 DEGs), the regulation of cellular processes (GO:0050794; 62 DEGs), and multicellular organismal processes (GO:0032501; 49 DEGs) (Figure 2B). These findings emphasize the regulatory roles of VIS in shaping cellular behaviour and coordinating complex biological processes. Enrichment in the ontology terms by chosen DEGs is visualized in Figure 3. A KEGG database analysis revealed one signalling pathway (Figure 4), neuroactive ligand–receptor activation (KEGG:04080; seven DEGs) (Supplementary Table S3). This comprehensive analysis provides a deeper understanding of how VIS influences gene expression, paving the way for further exploration of its molecular mechanisms.

### 2.3. Long Noncoding RNA Identification and Functional Annotations

We identified a total of potential 767 lncRNAs, including 302 lncRNAs described in the ENSEMBL database as “lncRNA”. The coding potential for the remaining unknown 465 lncRNA sequences was estimated using CPC [35] and CNCI [36]. The sequences of 160 alignments survived and were considered novel lncRNAs and subdivided into five class codes by StringTie [37]. For both revealed (known and novel lncRNAs) datasets, we performed a differential expression analysis. As a result, we obtained 40 DELs in the VIS-treated group, of which 3 lncRNAs were known and 37 were found to be novel. In this group, 23 (1 known and 22 newly identified) lncRNAs were upregulated and 17 (2 known and 15 newly identified) lncRNAs were downregulated in the presence of VIS. The calculated log_2_

FC values of DELs ranged from −22.21 (*MSTRG.10127.1*) to 23.72 (*MSTRG.579.3*) (Supplementary Table S4).

The cis-interaction analysis revealed a total of 327 DELs. In a further analysis, we used the custom Python script to detect messenger RNA (mRNA)–lncRNA binding. We identified 1898 relationships in mRNA-DEL expression, but only 69 of them were statistically significant and connected with five DELs. This dataset was used to estimate the Pearson correlation. We identified 37 interactions with negative correlations and 28 events with positive correlations. However, the correlations were not strong enough to use the data in a further analysis, and we did not identify any significant DEL–DEG pair. Therefore, we do not include data on DEL–DEG interactions later in this article. In the functional analysis, we focused on the significant DEL–target gene interactions.

**Figure 5:**
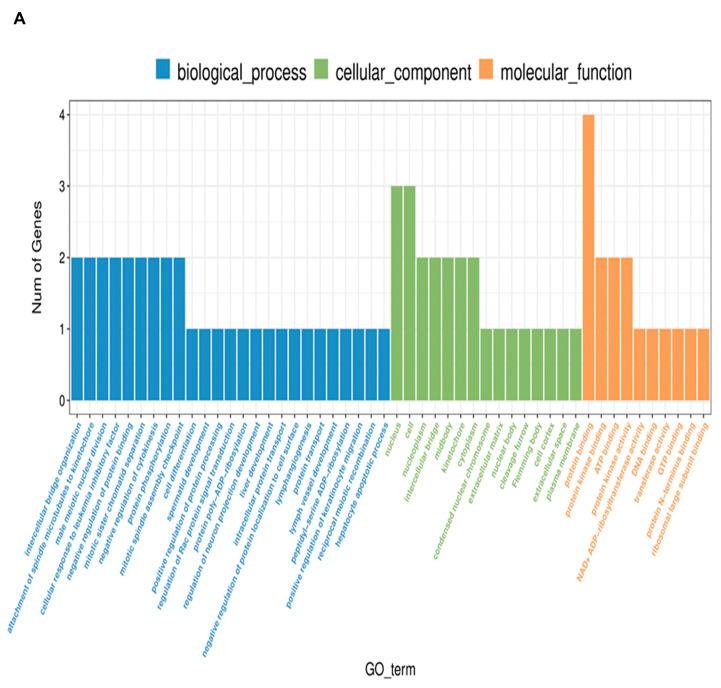

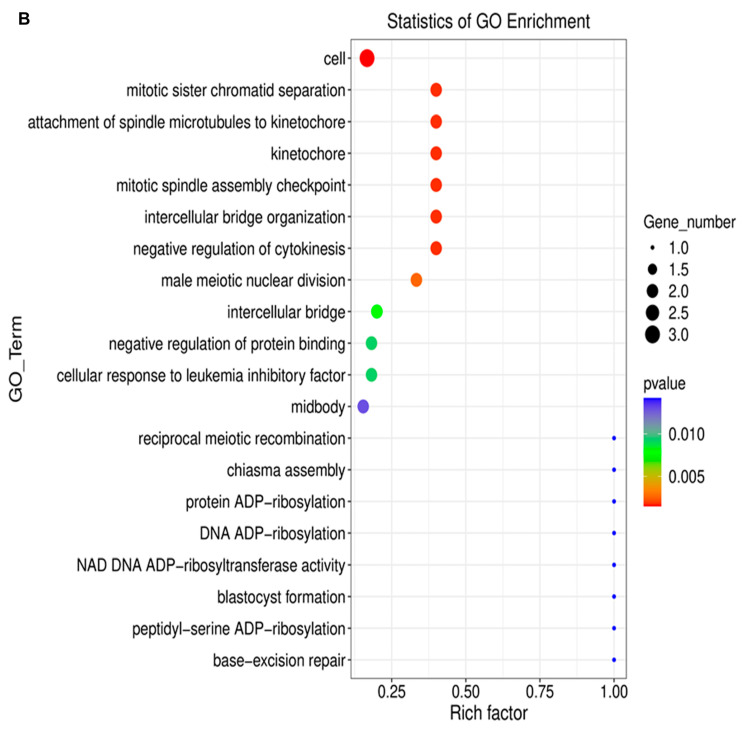
Summary of enriched Gene Ontology (GO) components of lncRNA-target genes evaluated in the luteal cells treated with visfatin. (**A**) The bar plot shows GO enrichment terms in three categories: biological process (BP), cellular components (CC), and molecular function (MF). The length of the bar represents of numbers of lncRNA target genes (Y-axis) annotated to GO terms (X-axis) (**B**) Dot plot representing results based on the differentially expressed lncRNA target genes in enrichment in the ontology terms. The colour of the dots represents statistical significance. The size of dots visualises the number of lncRNA target genes annotated to GO terms. The Y-axis and X-axis correspond to enriched GO terms and the value of their enrichment, respectively.

To further investigate the potential function of DELs, a GO functional term enrichment analysis and a KEGG signalling pathway enrichment analysis were also performed. A total of 68 differential expressions of cis-acting DELs were obtained following the conversion of the transcript ID into the gene ID. To further reveal the biological pathway information of the potential target genes of these DELs in LCs of the VIS-treated group, a GO functional term enrichment analysis was performed. Representative GO terms significantly enriched by DELs were identified based on the *p*-value (*p* ≤ 0.001, manually removing redundant functional terms, Figure 5A). The functional analysis demonstrated that the cis-target genes of DELs were enriched in 31 GO terms in three categories of BP, CC, and MF (Figure 5B). The corresponding potential target genes were subjected to a KEGG signalling pathway enrichment analysis. These target genes were mapped to seven signalling pathways; one of them was statistically significant (*p* < 0.05): base excision repair (map03410). Therefore, two mRNAs were selected—collagen and calcium binding epidermal growth factor domains 1 (*CCBE1*) and cyclin B1 interacting protein 1 (*CCNB1IP1*)—which, among others, enriched the positive regulation of angiogenesis and blastocyst formation. Significant differences were not discovered in the expression levels of mRNAs, but only in the expression of the corresponding lncRNAs (log_2_FC ± 0.56, *p* ≤ 0.01).

### 2.4. Alternative Splicing Event Analysis

**Figure 6:**
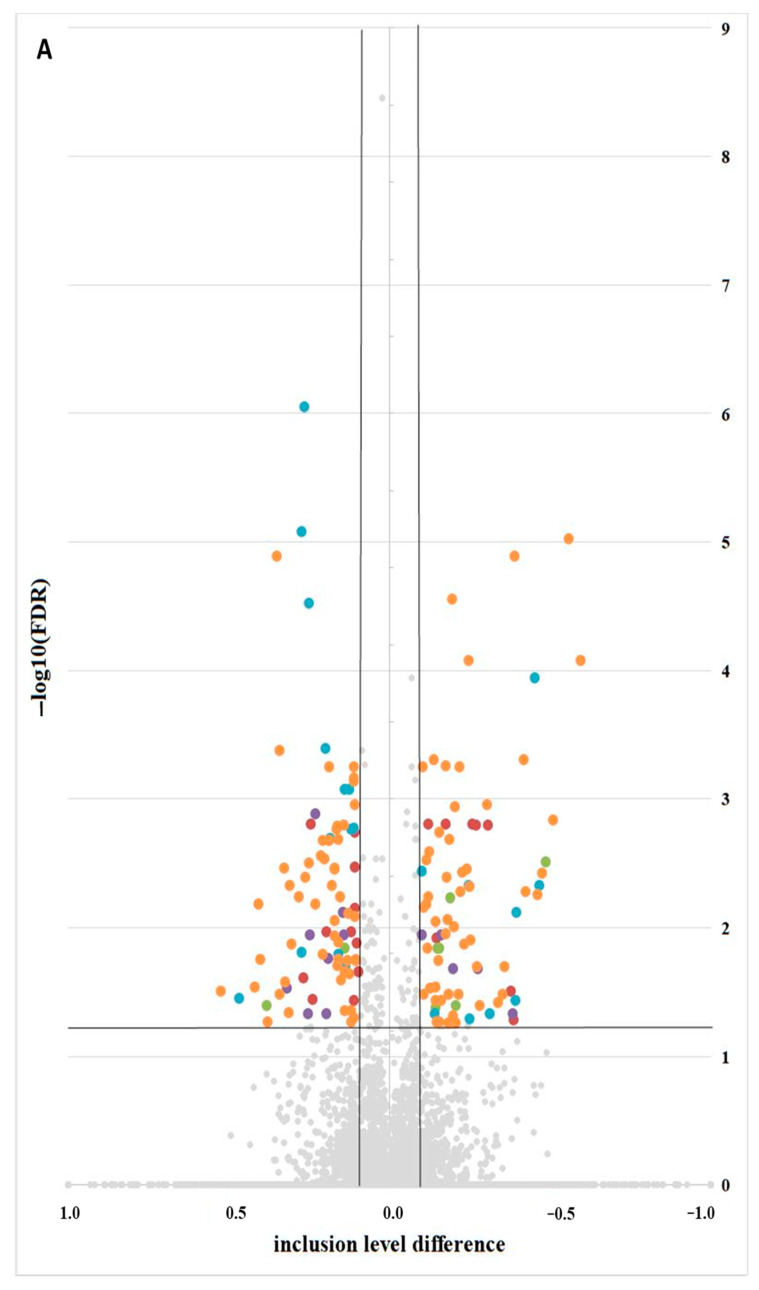

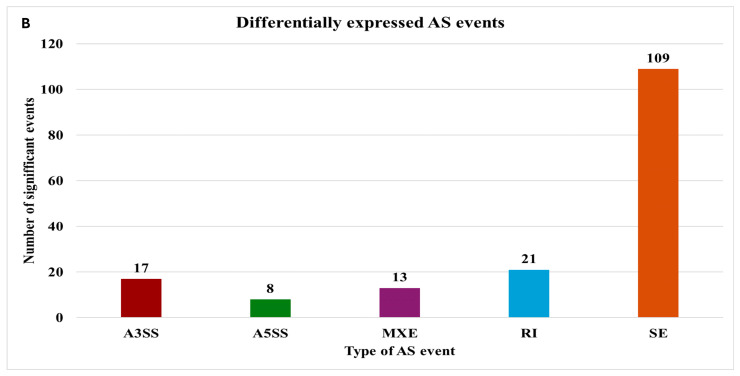
Visualization of alternative splicing events in response to visfatin (VIS). (**A**) Volcano plot presenting inclusion level of differentially alternative splicing events (DASs). Black lines indicate the cut-off thresholds, described in the text. The coloured dots represent the different types of significant DASs (*p* < 0.05). Inclusion levels of each DASs are presented on the X-axis, and the negative logarithmic adjusted *p* value is presented on the Y-axis. (**B**) Bar plot showing the appearance of DASs. Abbreviations: AS—alternative splicing event, A3SS—alternative 3′ splice site (red), A5SS—alternative 5′ splice site (green), MXE—mutually exclusive exon (purple), RI—retention intron (blue), SE—skipping exon (orange), not sig—not significant (grey).

To identify alternative splicing events (ASEs) and analyse differential alternative splicing events (DASs) between samples, we used the rMATS [38] software. We detected 37,460 ASEs, of which 168 DASs (Supplementary Table S5) were significant when comparing VIS-treated LCs to the control group (Figure 6A). The DASs were classified into five groups of ASE types: 17 as alternative 3′ splice sites (A3SSs), 8 as alternative 5′ splice sites (A5SSs), 13 as mutually exclusive exons (MXEs), 21 as retention introns (Ris), and 109 as skipping exons (SEs) (Figure 6B). The estimated inclusion level difference values ranged from −0.594 (*FBXO15*; SE) to 0.527 (*YAF2*; SE). All identified DASs were localized within 149 genes and were not correlated with DEGs or DELs. DASs were identified within the genes involved in the synthesis of glycosylphosphatidylinositol (GPI), PIGN, and RECK. PIGN is a crucial enzyme in the one of steps of the GPI-anchor biosynthetic, whereas RECK is a GPI-anchored inhibitor of matrix metalloproteinases. In the case of the *PIGN* gene, we observed double AS-SE (*PIGN*; ΔPSI= −0.247; SE and ΔPSI= −0.165; SE) in VIS-treated cells. The *RECK* gene showed two types of ASEs—first, SE (ΔPSI= −0.099), and second, MXE (ΔPSI= −0.107)—in the same experimental group. Consequently, VIS administration was related to the more frequent MXE occurrence in the range of *RECK*. Within the signalling pathways regulating the pluripotency of stem cells, we identified two genes, *CPSF7* and *FBXO15*. There was one ASE for each gene (*CPSF7*; ΔPSI= −0.193; SE and *FBXO15*; ΔPSI = −0.594, respectively; SE). The analysis showed three ASEs, among which two were more strongly expressed in the VIS-treated group (ΔPSI = 0.214; SE and ΔPSI = 0.126; SE). Among significant DAS genes playing a key role in the proliferation, apoptosis, cell cycle, and key regulation of DNA replication initiation in eukaryotes, the *CDC45* gene indicates the changes in inclusion level difference (ΔPSI = 0.111) in the VIS-treated group.

### 2.5. Validation of RNA-Seq Results

**Figure 7:**
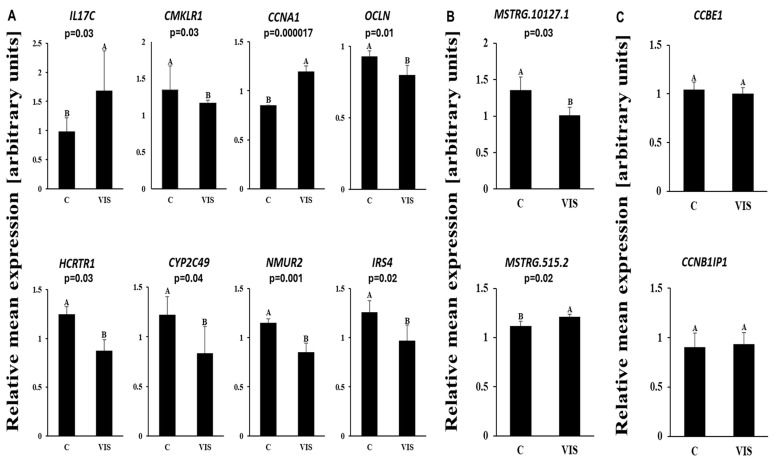
(**A**) Quantitative real-time PCR validation of RNA-seq results for DEGs in the luteal cells. Validation was performed for IL-17C, CMKLR1, CCNA1, OCLN, HCRTR1,CYP2C49, NMUR2, and IRS4 genes with reference genes (GAPDH—glyceraldehyde-3-phosphate dehydrogenase; ACTB—actin) (*p*-value < 0.05). (**B**) Quantitative real-time PCR validation of the RNA-seq results for lncRNAs in the luteal cells. Validation was performed for lncRNAs (MSTRG.515.2 and MSTRG.10127.1) and (**C**) their corresponding target genes, CCBE1 and CCNB1IP1, with reference genes GAPDH and ACTB (*p*-value < 0.05). Abbreviations: C—control group; VIS—visfatin-treated group; IL-17C—interleukin 17C; CMKLR1—chemokine-like receptor 1; CCNA1—cyclin A1; OCLN—occludin; HCRTR1—hypocretin receptor 1; CYP2C49—cytochrome P450 family 2 subfamily C member 49; NMUR2—neuromedin U receptor 2; IRS4—insulin receptor substrate 4; CCBE1—collagen and calcium binding epidermal growth factor domains 1; CCN1IP1—cyclin B1 interacting protein 1; A/B—statistically difference between groups.

**Figure 8:**
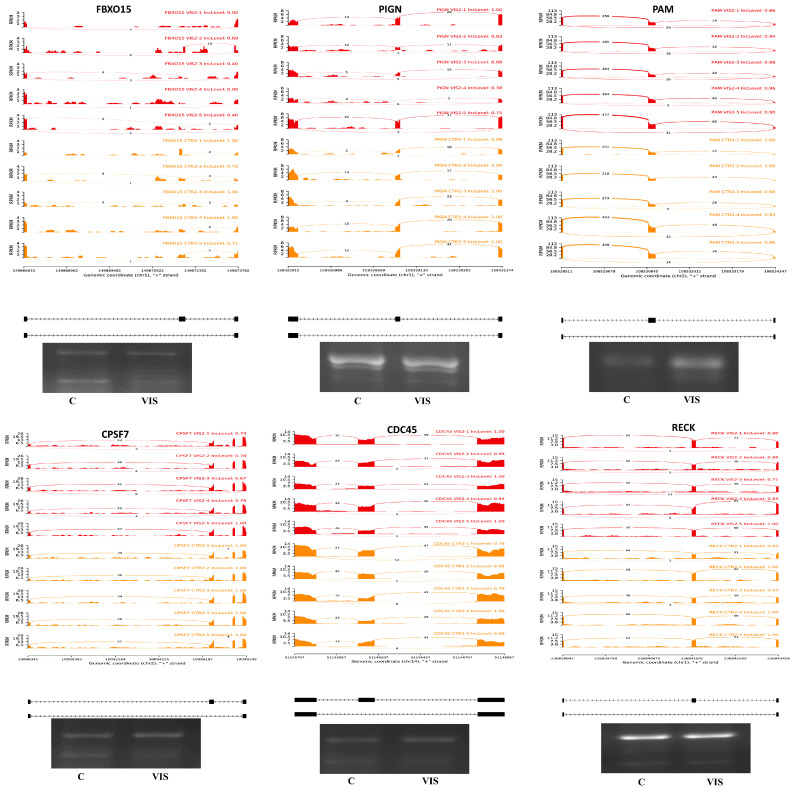
Quantitative visualisation (Sashimi plot) of differential alternative splicing events (DASs) statistically significant in the changes in percentage splicing inclusion (adjusted *p*-value < 0.01 and |ΔPSI| > 0.1) between visfatin-treated group (VIS; red) and control group (C; yellow/orange) samples. The numbers in each upper-right corner of the red and yellow/orange tracks present the percentage of splicing inclusion (PSI) values. Splicing differentiations are covered by the number of reads mapped in the range of junction sites. The upper black tracks show the genomic localisation of splicing events. The plot was generated in ggsashimi Python script. Below the plots, we present the validation results of chosen DASs by using semiquantitative PCR. The images show the inclusion and skipping exon levels between VIS and C groups. Abbreviations: FBXO15—F-Box protein 15; PIGN—phosphatidylinositol glycan anchor biosynthesis class N; PAM—peptidylglycine alpha-amidating monooxygenase; CPSF7—cleavage and polyadenylation specific factor 7; CDC45—cell division cycle 45; RECK—reversion inducing cysteine-rich protein with Kazal motifs.

To validate the obtained RNA-Seq results, the expression of eight DEGs (interleukin 17C—*IL17C*; chemokine-like receptor 1—*CMKLR1*; cyclin A1—*CCNA1*; occludin—*OCLN*; hypocretin receptor 1—*HCRTR1*; cytochrome P450 family 2 subfamily C member 49—*CYP2C49*; neuromedin U receptor 2—*NMUR2;* and insulin receptor substrate 4—*IRS4*) as well as two DELs (*MSTRG.515.2* and *MSTRG.10127.1*) and their target genes (*CCBE1* and *CCNB1IP1*) were analysed using RT-qPCR. In the LCs VIS-treated group, compared to the control group (n = 5), the relative mRNA expression of *IL17C*, *CMKLR1*, *CCNA1*, and *MSTRG.515.2* was upregulated, whereas the mRNA levels of *OCLN*, *HCRTR1*, *CYP2C49*, *NMUR*, *IRS4,* and *MSTRG.10127.1* were downregulated. The tendency of inclusion/exclusion differences within six DASs (*FBXO15*, *PIGN*, *PAM*, *CPSF7*, *CDC45*, and *RECK*) between the VIS-treated and control groups was confirmed by the PCR validation procedure. The administration of VIS was related to a higher frequency of ASEs in the range of *RECK*, *FBXO15*, and *PAM*. In contrast, the validation of the *CDC45*, *PIGN*, and *CPSF7* genes revealed, more frequent ASEs in the control group. The expression patterns of the selected DEGs, DELs, and tendency calculations of the exon inclusion/exclusion of DASs agreed with the RNA-Seq results (Figure 7A–C and Figure 8). A visualization of the RNA-Seq pattern expression is shown in Supplementary Figure S2. The validation of the results confirmed the veracity and accuracy of the high-throughput methods used in the present study.

## 3. Discussion

Pituitary-derived gonadotrophins and growth hormone are the primary regulators of LCs’ function in domestic animals, including pigs [11,39,40]. However, there are several endocrine factors that play a vital role in the regulation of LCs’ function in endocrine, autocrine, paracrine, and juxtracrine manners. The sparse literature reports show that hormones produced by the adipose tissue can play a crucial role in the process of fertilization and reproduction [32]. VIS has been reported to be the adipokine potentially involved in the control of fertilization and reproduction as well as steroidogenesis processes [17,19,41]. The effect we discovered of VIS on these processes in porcine LCs [34] was the basis for the hypothesis that the adipokine influences the transcriptome of these cells. The knowledge on VIS’s impact is primarily associated with understanding the mechanism of the adipokine’s action and its involvement in the biological processes of LCs. The present study was motivated by the lack of data on the transcriptomic profile of porcine LCs under the impact of VIS. It is also the first study that demonstrated the effect of VIS on ASEs and lncRNAs occurring in LCs.

In this study, we confirmed the stimulatory impact of VIS on the expression of two genes: cyclin A1 (*CCNA1*) and androgen-dependent tissue factor pathway inhibitor regulating protein (*ADTRP*). The cyclins modulate transcription and are key regulators of cell cycle progression, communication, and apoptosis [42,43,44]. The product of *CCNA1* is predicted to play a regulatory role in the control of the cell cycle, especially at the beginning of the S-phase and the end of the M-phase [42,43,45]. The expression of *CCNA1* in the gonads of mammals can positively regulate its function, affecting mammalian reproduction [45]. Kfir et al. [46] confirmed the expression of *CCNA1* in bovine ovaries during the early- and mid-luteal phases of the oestrous cycle. Yoshioka et al. [42] showed that cyclin genes are more strongly expressed in small LCs than in large LCs. The latest report suggests that CCNA1 is engaged in the oocyte and early embryo development during embryogenesis [41]. The phosphorylation of Akt in the absence of CCNA1 could lead to G2/M meiotic arrest and signalling to enter apoptosis because of defective meiosis in the male germ cells [45]. Moreover, we identified changes in lncRNA expression whose target gene is cyclin B1 interacting protein 1 (*CCNB1IP1*). CCNB1IP1 is related to the G2/M cell cycle checkpoint regulator, coordinating the cell cycle, migration, and invasion [47]. Its overexpression can lead to tumour development [48]. Toby et al. [49] showed that this protein can interact with cyclin B1 and promote its degradation. So far, little is known about the interaction of lncRNA-*CCNBIP1*. ADTRP, the prosurvival factor, promotes cell proliferation correlated with cyclins and inhibits apoptosis by decreasing the expression of some apoptosis-related genes or suppressing histone expression, as well as increasing the number of S-phase cells during the cell cycle [44]. CCNA1 and ADTRP can impact these cellular processes via the Akt pathway [45,50]. In the developing CL, along with steroidogenesis, robust angiogenesis takes place [51]. CCNA1 and ADTRP are also associated with the cell migration [43,44] as well as angiogenesis [43,44], and both may be involved in many signalling pathways, i.a. those related to vascular endothelial growth factor (VEGF), a key regulator of angiogenesis [52,53]. Bauer et al. [53] reported that *CCNA1* is a valid target for haem oxygenase-1. This interaction promotes angiogenesis [53]. *ADTRP* mRNA levels are highly increased during embryogenesis [54]. In vivo studies indicated that ADTRP plays a crucial role in embryogenesis as well as vascular development, stability, and integrity. *ADTRP* knock-out in mice is mostly lethal, and survived newborns showed a wide spectrum of vascular defects [54]. The proposed clarification of the disorder’s mechanism is the temporally aberrant Wnt signalling [29,54]. The effect of VIS on angiogenesis has also been confirmed [29,55,56], and it cannot be ruled out that one of the mechanisms of its action may be the effect on CCNA1 and ADTRP. During the analysis, we also identified the downregulation of lncRNA *MSTRG.515.2.*, whereas its target gene, collagen and calcium-binding epidermal growth factor domain-containing protein 1 (*CCBE1*), did not show any change in expression. CCBE1 is responsible for extracellular matrix remodelling and migration [57]. This gene is also required for lymphangiogenesis [58,59]. Moreover, *CCBE1* is engaged in blastocyst formation [60,61]. It is interesting that only lncRNA expression was downregulated as an impact of VIS, while mRNA did not show any changes in expression. The GO analysis ascribed both genes, *ADTRP* and *CCBE1*, as crucial factors in the cell migration involved in angiogenic sprouting from the venous endothelium during embryogenesis. Disorders in their expression during embryogenesis can lead to lethal defects [54,62]. It is possible that VIS, by stimulating the expression of *CCNA1* and *ADTRP,* may be involved in the regulation of the cell cycle, promoting cell proliferation as well as tissue remodelling and the development of the CL, which consequently creates a fully physiologically functional organ, able to synthesize the steroid hormones and maintain pregnancy. It seems that VIS, by the regulation of CL remodelling and the development of ovarian vasculature, may be an important factor in providing the correct endocrine interaction between the pituitary, ovaries, and uterus.

The control of reproductive processes is related to the regulation of food intake and energy homeostasis by the hormones and neuromodulators of the central nervous system. In our study, we showed an increase in chemokine-like receptor 1 (*CMKLR1*) gene expression, the gene coding the main receptor for chemerin (CHEM), as well as a lower content of orexin receptor 1 (*HCRTR1*) mRNA in VIS-treated LCs. The expression of CMKLR1 was described in the various tissues, including the hypothalamus, pituitary, ovaries, uterus, trophoblasts, and conceptuses [63,64,65,66]. Our previous study showed that CHEM stimulated VIS expression in the uterus of pregnant pigs [7]. CHEM and its receptors (chemerin system) are engaged in inflammatory processes, the promotion of angiogenesis and adipogenesis, metabolic diseases, and the regulation of energy balance, as well as participating in the control of ovarian function [67,68,69]. We have indicated that CHEM affects porcine ovarian steroidogenesis by the modulation of P_4_, androstenedione (A_4_), testosterone (T), oestrone (E_1_), and oestardiol (E_2_) secretion. In porcine LCs, CHEM treatment altered the expression of biomarkers related to angiogenesis and apoptosis [70]. The regulatory effects of CHEM on the CL in pigs were further verified by the transcriptomic analysis, which also showed that the adipokine takes part in the processes of steroid and prostaglandin synthesis, inflammatory responses, and regulating the secretion of luteotropic and luteolytic signals [9,10]. Moreover, we found a relationship between the expression of *CMKLR1* and *VIS,* which is indicated by the mentioned increase in VIS mRNA content under the influence of CHEM as well as *CMKLR1* expression under the influence of VIS. GO assigned *CMKLR1*, among others, to the following GO terms: GO:0004930 (G protein-coupled receptor activity), GO:0005887 (integral component of plasma membrane), GO:0006954 (inflammatory response), GO:0007165 (signal transduction), and GO:0007186 (G protein-coupled receptor signalling). We suppose that VIS can interact with various adipokines. Its effects on the expression of adipokines and potential mutual modulatory properties may be an exciting topic for our further research.

Orexin A (OXA) binds selectively to the HCRTR1 receptor. The presence of orexin receptors was confirmed in the ovaries of different species, including pigs [71]. OXA takes part in the regulation of food intake, energy metabolism, and reproductive functions, and can modulate the endocrine function of HPG and the hypothalamic–pituitary–adrenal (HPA) axes [72,73]. Our earlier research showed the uterine expression of HCRTR1 and orexin receptor type 2 (HCRTR2) and the impact of OXA on steroidogenic enzyme content as well as P_4_ and E_2_ secretion in the porcine uterus [69,74]. The impact of orexins on steroid secretion by the porcine granulosa, theca interna, and LCs was also evidenced [75]. Ciccimarra et al. [76] showed that basal E_2_ production by the porcine granulosa cells is increased after OXA treatment. It is also suggested that OXA and B inhibited FSH-induced E_2_ secretion by pig granulosa cells [75]. In turn, Basini et al. [77] showed that OXA inhibited P_4_ production by the CL. In our previous study [71], we indicated that prepro-orexin gene expression and the presence of OXA and OXB vary according to the animal’s hormonal status. Silverya et al. [78] indicated that orexinergic blockers had an inhibitory effect on the gonadotrophins’ secretion and ovulation. Moreover, orexins regulate the neuroendocrine and behavioural responses that are affected by stress, including HPA axis disruption [79]. Elevated orexin levels and elevated HPA responses were observed in female rats under repeated stress [79]. The adaptation to pregnancy is modulated by endocrine signals like the elevated concentrations of P_4_ and E_2_, but also glucocorticoids (GCSs). The interactions between GCSs and sex hormones are unclear but might be related to pregnancy maintenance and foetal development. Stress-induced GCSs may directly influence P_4_ synthesis, as GCS receptors are also expressed in the ovaries [80]. GCSs and P_4_ have a common steroidogenic pathway and precursor such as pregnenolone. The synthesis of cortisol induced by stress decreased the availability of pregnenolone to P_4_ synthesis [80]. Furthermore, orexins increased plasma concentrations of aldosterone and corticosterone in rats and induced their secretion in the adrenocortical cells [81]. These limited studies suggest the novel physiological role of OXA/HCRTR1 signalling in regulating the female reproductive system. Similarly to *CMKLR1*, the *HCRTR1* gene, encoding OX1R protein, was assigned, among others, to the following GO terms: GO:0004930 (G protein-coupled receptor activity) and GO:0007186 (G protein-coupled receptor signalling pathway). Moreover, *HCRTR1*, as well as *NMUR2*, enriched GO:0007218 (neuropeptide signalling pathway) and neuroactive ligand–receptor activation (KEGG:04080), which is shown in Figure 4. Previous transcriptomic studies on pigs revealed that neuroactive ligand–receptor activation interaction may affect reproduction processes [82]. In our study, we indicated a decreased expression of *HCRTR1* in VIS-treated LCs. This can be linked with the defensive mechanism against stress, which may reduce P_4_ levels during pregnancy, and as a consequence, may lead to pregnancy complications and/or the loss of embryos. Our study implies that VIS may play an important role in female reproduction through the regulation of the orexigenic system in the ovaries.

As mentioned above, the CL consists of various cells, including endothelial cells (ECs). These cells are responsible for controlling vascular permeability, which plays a crucial role in the development of the CL. Permeability is controlled by the regulated opening and closing of the cell-to-cell contacts between ECs [83]. Defects of the adhesive junction (AJ) and tight junction (TJ) can lead to blood vessel perturbances. These junctions are necessary for controlling the paracellular endothelial permeability, promoting cell contacts, and transmitting intracellular signals [84]. It is not surprising that cell adhesion molecules are expressed in the CL. In our research, we showed the downregulation of occludin (*OCLN*) gene expression. OCLN is important in TJ stability and barrier function [83,84]. The GO analysis assigned this gene to GO:0005911 (cell–cell junction) and GO:0005923 (bicellular tight junction). In the human ovaries, during the normal luteal phase, it can be localised in the plasma membranes of ECs and luteinising granulosa cells. In humans, after the in vivo application of human chorionic gonadotropin (hCG), a significant decrease in OCLN in the luteinizing granulosa cells and ECs was observed. The authors suggest the hormonal control of cell junctions in the CL, which might be associated with tissue remodelling and increased luteal permeability during early pregnancy [85]. OCLN is essential for mediating cell–cell adhesion between adjacent LCs and is crucial for maintaining the overall architecture of the CL [85]. These tight junctions create a barrier that regulates the passage of ions, molecules, and fluids between LCs. OCLN’s role in maintaining the barrier function and structural integrity of LCs is indirectly linked to hormone secretion. The disruption of tight junctions and OCLN function can affect the release of hormones. It is possible that a decreased expression of cell adhesion molecules during the luteinisation process can be related to the release of steroidogenic hormones, including P_4_, and angiogenesis-related VEGF [86]. Rescuing the CL from its programmed senescence maintains the P_4_ production required for pregnancy maintenance and is characterized by a new structural orientation. An increase in the intercellular gaps promotes the expansion and invasion of new vessels and the transmissibility of the endothelium. The level of OCLN expression in the membranes of luteinising granulosa cells of monkeys decreases prior to the ovulation phase, after which it is no longer detected [87]. The decrease in *OCLN* expression seems to be involved in the formation of the antrum [88,89]. In contrast, VEGF antagonist administration leads to an increase in OCLN phosphorylation [89]. The downregulation of this adhesive factor by hCG and VEGF may lead to increased endothelial permeability and the release of steroid hormones into the blood. Our result might indicate that VIS, by suppressing the expression of *OCLN*, has a positive effect on remodelling and vascularization in CL, and thus, increased secretion of steroids from ovaries to the bloodstream, which is necessary for the physiological course of pregnancy.

The current study provides new information on the AS of transcripts in the porcine LCs treated with VIS. DASs were observed for *PIGN*, *RECK*, *PAM*, and *CDC45*. *PIGN* is involved in GPI-anchor biosynthesis and *RECK* is associated with the cell membrane through GPI-anchors. Mutations in the *PIGN* sequence cause the low expression of GPI-anchored proteins (GPI-APs) that are involved in cell adhesion, signal transduction, and antigen presentation. This results in global development delay, minor dysmorphic features, congenital anomalies, and many other disorders [90]. In the embryos, the biallelic truncation of *PIGN* leads to a loss of function and early death in utero or soon after birth [90]. RECK is a key regulator of extracellular matrix integrity and angiogenesis. Furthermore, the short form of *RECK* may interact with its long form and interfere with matrix metallopeptidase 9 (MMP-9), resulting in the regulation of the cell migration rate [91,92]. The expression of *RECK* mRNA was observed at all stages of ovarian tissue remodelling, during folliculogenesis, the ovulation–osteogenic transition phase, CL maintenance, and regression [93]. Mammals have a single *PAM* gene encoding multifunctional proteins. PAM, a copper-dependent enzyme, is the only mediator for producing the plethora of amidated peptides involved in homeostasis, embryogenesis, and the control of thyroid hormone levels. In the anterior pituitary, sex steroids and glucocorticoids regulate *PAM* mRNA concentrations and protein activity [94]. The genetic elimination of *PAM* results in embryonic lethality. *PAM*-null mouse embryos do not survive embryonic day 15. The rising P_4_ production associated with ovulation may be influenced by PAM. Its role may include fine-tuning of P_4_ production and cellular regression [95]. CDC45 takes part in the initiation of DNA replication related to the cell cycle, mitotic and chromatin binding, and DNA replication [96]. The downregulation of *CDC45* arrests cells in the G1 phase and represses the expression of G1-/S-phase-related genes [97]. *CDC45* homozygous mutant mice die in the early embryonic stage due to the impaired proliferation of inner cell mass [97]. Mutations in *CDC45* can be related to a wide spectrum of genetic disorders [97]. The results allow us to assume that VIS can be engaged indirectly in controlling the cell cycle processes, ovarian tissue remodelling, cell migration, CL maintenance, and regression, as well as embryo development, by preventing their death in the early stages of pregnancy. This suggests that VIS can modulate AS processes and thus play a regulatory role in LCs. Further research is needed to understand the physiological meaning of the noted DASs.

## 4. Materials and Methods

### 4.1. Collection of Samples

The use of animals was in accordance with the Polish Act of 15 January 2015 on the protection of animals used for scientific or educational purposes (Journal of Laws, 2015, item 266) and Directive 2010/63/EU of the European Parliament of 22 September 2010 on the protection of animals used for scientific purposes, and the experiments did not require approval from the ethical commission. The experimental animals were female cross-bred mature gilts (n = 5, Large White × Polish Landrace, aged 7–8 months and 130–150 kg in body weight), obtained from the breeding farm in Balcyny, belonging to the University of Warmia and Mazury in Olsztyn, Poland. The gilts were observed daily for oestrus behaviour in the presence of a boar. The day of the onset of the second oestrus was marked as day 0 of the oestrous cycle. Mating of the pigs was performed on days 1–2 of the oestrous cycle. The next day after the mating was denoted as the first day of pregnancy. Ovaries were collected from the females on days 15–16 of pregnancy (the beginning of implantation), and immediately after slaughter, they were transported to the laboratory in ice-cold PBS supplemented with 100 IU/mL of penicillin and 100 µg/mL of streptomycin. The day of pregnancy was also confirmed by ovarian morphology [98] and additionally evaluated by the presence, size, and morphology of conceptuses collected from both uterine horns [99].

### 4.2. In Vitro Culture of Luteal Cells and Total RNA Isolation

The LC isolation procedure and in vitro culture were performed according to Rytelewska et al. [70] with modification. Luteal cells were enzymatically dispersed (4–6 times) in 0.1% collagenase type V in Hank’s balanced salt solution (Sigma-Aldrich, St. Louis, MO, USA). Isolated cells were centrifuged (300× *g*, 10 min, 37 °C) and washed three times with sterile F-12 medium (Sigma-Aldrich, St. Louis, MO, USA). To remove non-dispersed cell clumps, a sterile nylon filter (70 μm mesh; Greiner, Kremsmünster, Austia) was applied. The cells were counted, and their viability was determined by the trypan blue (Sigma-Aldrich, St. Louis, MO, USA) dye exclusion method. The mean percentage of viable cells after isolation was 96.76% ± 1.3%. The LCs were resuspended to a concentration of 2 × 10^6^/2 mL in F-12 medium with 10% foetal bovine serum (Sigma-Aldrich, MO, USA), 0.1% bovine serum albumin (MP Biomedicals, Irvine, CA, USA) and 1% antibiotic-antimycotic solution (Sigma-Aldrich, St. Louis, MO, USA) in 6-well culture plates. The cells were preincubated for 48 h in a humidified incubator (37 °C; 95% air, 5% CO_2_). After preincubation, the media were removed. New serum-free media were added and the cells were treated for 24 h with recombinant human VIS (R&D System, Minneapolis, MN, USA) at a concentration of 100 ng/mL or without any treatment (n = 5). The VIS was diluted in free FCS and BSA F12 medium to the appropriate concentration. The concentration of VIS was selected based on Reverchone et al. [19]. After in vitro culture, from LCs treated and untreated with VIS, total RNA was extracted with the use of Extrazol (BLIRT, Gdansk, Poland), following the manufacturer’s procedure. The purity (A260/A280) and quantity (wavelength 260 nm, A260) of the obtained RNA were estimated spectrophotometrically with the Infinite M200 Pro (Tecan, Männedorf, Switzerland). RNA integrity was tested using the Bioanalyzer 2100 (Agilent Technology, St. Clara, CA, USA). Only samples with purity (A260/280) > 1.8 and an RNA integrity number (RIN) > 8 qualified for RNA-Seq analysis as well as the polymerase chain reaction (PCR) and quantitative real-time polymerase chain reaction (qPCR) validations. The workflow of the procedure of the performed analysis is depicted in Supplementary Figure S1. The viability of the cells, as well as the potential toxicity of the treatment, were determined using the AlamarBlue™ (Thermo Fisher Scientific, Waltham, MA, USA) assay according to the manufacturer’s instructions. The average reduction of AlamarBlue™ in cultured LCs after the treatment period was 107.93 ± 2.91% in the VIS-treated group compared to the control group.

### 4.3. Library Construction and RNA Sequencing

The ribosomal RNA (rRNA) was removed according to the protocol of the Ribo-Zero Gold rRNA Removal Kit (Illumina, San Diego, CA, USA). The remaining RNAs were fragmented into short fragments using divalent cation buffers under high temperatures. The sequencing library was prepared following the TruSeq Stranded mRNA Library Prep Kit (Illumina, San Diego, CA, USA), according to the manufacturer’s protocol. Briefly, after long RNA molecules’ fragmentation, poly(dT) oligonucleotides were applied to the transcription of RNA into cDNA. Next, the double-stranded cDNA fragments were subjected to the end-repair and A-tailing processes and adaptor ligation. Finally, PCR amplifications were performed to enrich cDNA libraries. A quality control analysis and quantification of the sequencing library were performed using an Agilent Technologies 2100 Bioanalyzer High Sensitivity DNA Chip. Paired-ended sequencing was performed on Illumina’s NovaSeq 6000 sequencing system platform (Illumina, San Diego, CA, USA) to generate 2 × 150 nt paired-end reads with an assumed minimum sequencing depth of 40 million reads per sample. A global expression analysis was performed for the porcine LCs in five biological replicates for the VIS-treated group (n = 5) and control group (n = 5).

### 4.4. Bioinformatic Analysis

The genes’ expression as well as the lncRNAs’ and alternatively splicing (AS) transcripts’ production were analysed to investigate the regulatory mechanisms induced by VIS in the porcine LCs. The in silico analyses were mainly performed in accordance with Orzechowska et al. [7], Makowczenko et al. [100], and Paukszto et al. [101].

#### 4.4.1. Assembly of Transcripts and Processing of Differentially Expressed Transcripts

Firstly, Cutadapt ver. 1.9 [102] was used to remove the reads that contained adaptor contamination and low-quality scores (QPhred score ≤ 20; reads containing more than 5% of unknown nucleotides) and obtain high-quality clean reads. Then, the sequence quality was verified using the FastQC software, ver. 0.11.9 [103]. The resulting read sets of the analysed samples were mapped to the reference genome of *Sus scrofa domestica* (Sscrofa11.1. ver. 96) deposited in the ENSEMBL database [104] using HISAT2 ver. 2.0.4 [105]. The identification of the transcript active regions (TARs) was based on the assembly of the mapped reads of each sample, assembled using StringTie ver. 1.3.4d [37]. Then, transcriptomes from all 10 samples were merged to reconstruct a comprehensive transcriptome using gffcompare ver. 0.9.8 [37]. After the final transcriptome was generated, StringTie [37] and Ballgown ver. 2.30.0 [106] were used to estimate the expression levels of all transcripts. StringTie [37] was used to determine the levels of mRNAs and lncRNAs by calculating the fragments per kilobase of the transcript per million mapped reads (FPKM) value. A DEGs/DELs analysis was performed using the DESeq2 software, ver. 1.34.0 [107], between two different groups and using edgeR [108] between two samples. DEGs and DELs with *p*-value < 0.05 and a log_2_(FC) ≥ 0.56 were considered differentially expressed.

#### 4.4.2. Functional Annotation of Target Genes

The gene set enrichment analysis was performed using the GSEA software, ver. 4.1.0, [109] and the Molecular Signatures Database (MSigDB). Enrichment ontology and pathway analyses were performed with the use of the g:Profiler software [110] based on the Kyoto Encyclopaedia of Genes and Genomes (KEGG) [111] and Gene Ontology (GO) [112,113] databases. The gene expression matrix and rank genes were input by the Signal2Noise normalization method. The KEGG and GO terms with *p*-values < 0.05 were defined as significant.

#### 4.4.3. Long Noncoding RNA Identification, Target Gene Prediction, and Functional Analysis

To identify lncRNA candidates differentially expressed in the porcine LCs after VIS treatment, transcripts that overlapped with known mRNAs, known lncRNAs, and transcripts shorter than 200 bp were discarded. Then, we utilized the Coding Potential Calculator (CPC), ver. 0.9-r2 [35], and the Coding-Non-Coding Index (CNCI), ver. 2.0 [36], to predict transcripts with coding potential. All transcripts with a CPC score < 0.5 and a CNCI score < 0 were retained and considered as novel lncRNAs. To identify the new transcripts, all of the transcripts were reconstructed with StringTie and HISAT2 and were aligned to the reference genome using gffcompare [37,104,105]. Transcripts that did not match or overlapped annotated genes were labelled as ‘novel’ lncRNAs. Novel lncRNAs were subdivided into five categories according to their class code generated by StringTie [37]: (i) a transfrag falling entirely within a reference intron (intronic); (j) a potentially novel isoform or fragment, where at least one splice junction is shared with a reference transcript; (o) generic exonic overlap with a reference transcript; (u) unknown, intergenic transcript (intergenic); (x) exonic overlap with a reference on the opposite strand (antisense).

To explore the function of lncRNAs, we predicted the cis-target genes of lncRNAs. lncRNAs may play a cis role by interacting with neighbouring target genes. In this study, coding genes 100,000 bp upstream and downstream were selected using a custom Python script. Then, we conducted the enrichment analysis of GO functions and KEGG pathways of the target genes for lncRNAs. To identify DELs between the probes from the control and VIS-treated groups, the DESeq2 software was applied. Merging the DELs’ identification with DEGs, we collected DEGs/DELs modulated by the VIS treatment. Significance was expressed as a *p*-value < 0.05.

#### 4.4.4. Differential Alternative Splicing Event Analysis

A replicate multivariate analysis of the transcript splicing (rMATS) software, ver. 4.1.1 [38], was used to identify AS events and DASs between samples. Trimmed reads with a length of 120 bp were applied to estimate the percentage of splicing inclusion (PSI) in a range of splicing sites at intron/exon junctions. We identified DAS events with a false discovery rate (FDR), q-value < 0.05, and the absolute value of inclusion level difference, |ΔPSI| > 0.1, as significant.

### 4.5. Validation of DEGs and DELs by Quantitative Real-Time PCR (qPCR)

The qPCR method was performed to validate the selected DEGs and DELs with the use of the AriaMX Real-time PCR System (Agilent Technology, Santa Clara, CA, USA). Exactly the same RNA samples as used for the RNA-Seq were used for reverse transcription into cDNA with an Omniscript RT Kit (Qiagen, Germantown, MD, USA) according to the manufacturer’s protocol. The primers’ sequence for the reference genes, chosen DEGs, and DEL–mRNA pairs; the primer concentration; and the qPCR reaction conditions are shown in Supplementary Table S1. To verify the functions related to DELs in the VIS-treated LCs and control LCs, DEGs and DELs were selected based on the results of the lncRNA target gene prediction and enrichment analysis. Subsequently, several DEG/DEL pairs were screened using qPCR. The qPCR reaction mixture, at a final volume of 20 µL, consisted of 12.5 µL of Sensitive RT HS-PCR Mix SYBR (A&A Biotechnology, Gdansk, Poland), 0.24 µL of ROX (reference dye), cDNA (DEGs: 10 ng; DELs: 20 ng), forward and reverse primers, and RNase-free water. All reactions were performed in duplicate. As the reference genes, actin (*ACTB*) and glyceraldehyde-3-phosphate dehydrogenase (*GAPDH*) were chosen. The amplification specificity was examined at the end of the qPCR by melting curve analysis and/or gel electrophoresis of post-qPCR products. Calculation of the relative expression levels of validated DEGs and DELs was performed with the use of the comparative cycle threshold method (∆∆CT; [114]) and normalised using the geometrical means of the reference genes’ Ct values. The normality of the qPCR data distributions was confirmed using the Shapiro–Wilk test (*p* > 0.05) and the results were statistically checked by the Student t-test (*p* < 0.05) using the Statistica software (Statsoft Inc, Tulsa, OK, USA).

### 4.6. Validation of DASs by PCR

We performed validation of the DASs for the 6 chosen genes—F-Box protein 15 (*FBXO15*); phosphatidylinositol glycan anchor biosynthesis class N (*PIGN*); peptidylglycine alpha-amidating monooxygenase (*PAM*); cleavage- and polyadenylation-specific factor 7 (*CPSF7*); cell division cycle 45 (*CDC45*); and reversion-inducing cysteine-rich protein with Kazal motifs (*RECK*)—using Labcycler 48 s (Syngen Biotech, Wroclaw, Poland). The PCR reaction was carried out using the StartWarm HS-PCR Mix (A&A Biotechnology, Gdansk, Poland). The reaction mixtures, at a final volume of 25 µL, consisted of 12.5 µL of Hot Start PCR Mix, 400 nM of forward and reverse primers, 15 ng of cDNA, and nuclease-free water. The primer sequences and the conditions of the PCR reactions are described in Supplementary Table S6. The final amplicons were detected using 1.5% agarose gels with the addition of Midori Green Advance (Nippon Genetics Europe, Düren, Germany).

## 5. Conclusions

In conclusion, our study demonstrates that VIS takes part in the control of LCs’ function and regulates the expression of various genes, including those involved in the cell cycle, cell migration, remodelling, proliferation, and adhesion process, as well as angiogenesis, inflammation, and steroidogenesis. The above-presented data suggest that VIS may belong to key regulators of metabolic, immune, and reproductive processes occurring in the porcine CL. The loss of pregnancy and reduced fertility can be associated with the disruption of processes directly linked with this organ, as well as the interaction between ovaries and the uterus. We proved that VIS significantly affects the expression of many genes in the LCs of pigs, which is likely to result in the modification of physiological processes related to reproduction. Despite this, the various processes and interaction mechanisms of VIS’s impact are still unclear and require further studies.

## Data Availability

The data underlying this article are available in the European Nucleotide Archive database at https://www.ebi.ac.uk/ena and can be accessed with accession number PRJEB61451 (accessed on 19 April 2023).

## Supplementary Materials

The following supporting information can be downloaded at https://www.mdpi.com/article/10.3390/ijms25042339/s1. Ref [115,116,117,118,119,120,121,122,123] are cited in Supplementary Table S6.
