# Supplementary material for: Visfatin Affects the Transcriptome of Porcine Luteal Cells during Early Pregnancy
Source: Int J Mol Sci. 2024 Feb 16;25(4):2339. doi: 10.3390/ijms25042339 (PMC10889815; doi:10.3390/ijms25042339)
Supplement: Supplementary file 1 [file ijms-25-02339-s001.zip › Supplementary_Table_S6-sequences.pdf]

Table. Characteristics of primers and reactions conditions used in the study used to identify differentially expressed genes, long non-coding RNAs and alternatively spliced RNAs

Differential Gene Expression						
**Gene Symbol**	**Primers sequences**	**Primers (nM)**	**Amplicon Size**	**GenBank accession number**	**References**	**Reaction Conditions**
**IL17C**	F 5’-CTGGAAGCTGACACTCACG-3’<br>R 5’-GGTAGCGGTTCTCATCTGTG-3’	400	155 bp	XM_021220807.2	[115]<br>[116]	activation at 95°C - 10 min, followed by 40 cycles of denaturation at 95°C - 15 s, annealing at 60°C for - 1 min elongation - 72°C for - 1 min
**CYP2C49**	F 5’-<br>TCTCAACAGGAAAACGAATTTGTG-3’<br>R 5’-TATGGCTGAACCCACTTAAAATG-3’	200	153 bp	XM_013983597.2	[117]	
**IRS4**	F 5’-CCGACACCTCATTGCTCTTTTC-3’<br>R 5’-TTTCCTGCTCCGACTCGTTCTC-3’	200	73 bp	XM_006724713.4	[118]	
**CCNA1**	F 5’- GCGCCAAGGCTGGAATCTAT-3’<br>R 5’ – CCTCAGTCTCCACAGGCTAC-3’	200	196 bp	XM_005668339.3	[119]	
**NMUR2**	F 5’-CTCTGTTCTCTTCTCTCTGC-3’<br>R 5’- GCTTGATGACTGTACAGGTA-3’	200	107 bp	XM_003134131.3	[120]	
**OCLN**	F 5’- CAGTGGTAACTTGGAGGCGT -3'<br>R 5’- CCGTCGTGTAGTCTGTCTCG -3'	400	104 bp	NM_001163647.2	[121]	activation at 95°C - 10 min, followed by 40 cycles of denaturation at 95°C - 15 s, annealing at 57°C for - 1 min elongation - 72°C for - 1 min

**HCRTR1**	F 5’- ACCGCTGGTATGCCATCTACCAC-3′ R 5′-ATAAGGTCATCTGCCCAGCGTTCA-3′	400	27-49 (F) 208-232 (R)	AF097995	[71]	activation at 95°C - 10 min, followed by 40 cycles of denaturation at 95°C - 15 s, annealing at 59°C for - 1 min elongation - 72°C for - 1 min
**CMLKR1**	F 5’-GGACTACCACTGGGTGTTCG-3’ R 5’- GCCATGTAAGCCAGTCGGA-3’	400	174 bp	EU660866	[63]	
**ACTB**	F 5’-ACATCAAGGAGAAGCTCTGCTACG-3’ R 5’-GAGGGGCGATGATCTTGATCTTCA-3’	200	366 bp	U07786	[122]	
**GAPDH**	F 5’-CCTTCATTGACCTCCACTACATGGT-3’ R 5’-CCACAACATACGTAGCACCACGAT-3’	200	183 bp	NM_001206359.1	[123]	

Differential Alternative Splicing Events

**Gene Symbole**	**Primers sequences**	**Primers (nM)**	**Amplicon Size**	**GenBank accession number**	**References**	**Reaction Conditions**
**FBXO15**	F: 5’- TTCTACTTGGATGCTGTGACCT-3’ R: 5’ -GTCCACCATGATTGGCTGTG-3’	400	Inclusion 598	XM_005659828.3	herein	activation at 95°C - 10 min, followed by 40 cycles of denaturation at 95°C - 15 s, annealing at 58°C for - 1 min elongation - 72°C for - 1 min
			Skipping 387			
**CPSF7**	F: 5’- GCATCTTTCTTACCTTGTGCGTT-3’ R: 5’- TTTTCAGAGGCTACCACCACC-3’	400	Inclusion 460	XM_021082934.1		activation at 95°C - 10 min, followed by 40 cycles of denaturation at 95°C - 15 s,
			Skipping 306			

**PIGN**	F: 5'- AGCAGCTCTGGTCAGGTAAG-3'<br>R: 5'- TGATGGTCGGTGAGCATGTC-3'	400	Inclusion 884<br>Skipping 761	XM_021099768.1		annealing at 60°C for - 1 min, elongation - 72°C for - 1 min
**RECK**	F: 5'- AACCAAATGTGCCGTGATGT-3'<br>R: 5'- AGTTTGTGTGATGGCCTGCA-3'	400	Inclusion 352<br>Skipping 292	XM_021065979.1		
**PAM**	F: 5'- ATGGGACCGTGTTTGTTGGA-3'<br>R: 5'- GTCTGTGTGGGACTGTACACAT-3'	400	Inclusion 678<br>Skipping 292	XM_021084569.1		
**CDC45**	F: 5'- TGTCTAGGACCTGAGCGACA-3'<br>R: 5'- TGATGTGGTCTTTGCCACCAT-3'	400	Inclusion 551<br>Skipping 415	XM_021074124.1		
	lncRNA					
**Gene Symbole**	**Primers sequences**	**Primers (nM)**	**Amplicon Size**	**GenBank accession number**	**References**	**Reaction Conditions**
**CCBE1**	F: 5'- TGCTGGGATGTATTGGCGATT-3'<br>R: 5'- GCAACCGTCTGTCTTTGCTG -3'	300	140	AK395352.1	herein	activation at 95°C - 10 min, followed by 40 cycles of denaturation at 95°C - 15 s, annealing at 59°C for - 1 min, elongation - 72°C for - 1 min
**CCNB1IP1**	F: 5'- TATGCATGGGTCACTGCCTG -3'<br>R: 5'- CCGCACAGAACTCAGTCCAT -3'	300	142	XR_002345959.1		
**MSTRG.515.2**	F: 5'- AGCAGTTCAGTTTGGGCAGT -3'<br>R: 5'- GTAGAGTGGGCACATGGCTT -3'	300	135	XM_003121722.6		

**MSTRG.10127.1**	F: 5'- CATGTCCCTTGGGAAGGTCT -3'<br>R: 5'- GAGGTGAGTTCCCAGAGAACG -3'	300	150	XR_002345958.1		

ADTRP* -** androgen dependent TFPI regulating protein; *IL17C* - interleukin 17C; ***CYP2C49* -** cytochrome P450 2C49**;** ***LRP15* -** leucine rich repeat containing 3B; ***IRS4* -** insulin receptor substrate 4; ***CCNA1- cyclin a1; ***NMUR2* -** neuromedin U receptor 2; ***OCLN* -** occludin; ***HCRTR1* -** hypocretin receptor 1; ***CMLKR1* -** chemerin chemokine-like receptor 1; ***ACTB*** - actin beta; ***GAPDH*** - glyceraldehyde-3-phosphate dehydrogenase; ***FBXO15*** - ***CPSF7*** - cleavage and polyadenylation specific factor 7; ***PIGN*** - phosphatidylinositol glycan anchor biosynthesis class N; ***RECK***- reversion inducing cysteine rich protein with kazal motifs; ***PAM*** - peptidylglycine alpha-amidating monooxygenase; ***CDC45*** - cell division cycle 45 ***CCBE1***- collagen and calcium binding epidermal growth factor domains 1, ***CCNB1IP1***- cyclin B1 interacting protein 1, F – forward; R - reverse

Reference

115. Vella, G.; Ritzmann, F.; Wolf, L.; Kamyschnikov, A.; Stodden, H.; Herr, C.; Slevogt, H.; Bals, R.; Beisswenger, C. IL-17C Contributes to NTHi-Induced Inflammation and Lung Damage in Experimental COPD and Is Present in Sputum during Acute Exacerbations. *PLoS ONE* **2021**, *16*, https://doi.org/10.1371/JOURNAL.PONE.0243484.
116. Yamaguchi, S.; Nambu, A.; Numata, T.; Yoshizaki, T.; Narushima, S.; Shimura, E.; Hiraishi, Y.; Arae, K.; Morita, H.; Matsumoto, K.; et al. The Roles of IL-17C in T Cell-Dependent and -Independent Inflammatory Diseases. *Sci. Rep.* **2018**, *8*, 15750. https://doi.org/10.1038/s41598-018-34054-x.
117. Ramayo-Caldas, Y.; Mach, N.; Esteve-Codina, A.; Corominas, J.; Castelló, A.; Ballester, M.; Estellé, J.; Ibáñez-Escriche, N.; Fernández, A.I.; Pérez-Enciso, M.; et al. Liver Transcriptome Profile in Pigs with Extreme Phenotypes of Intramuscular Fatty Acid Composition. *BMC Genom.* **2012**, *13*, 547. https://doi.org/10.1186/1471-2164-13-547/FIGURES/6.
118. Cantarini, M.C.; De La Monte, S.M.; Pang, M.; Tong, M.; D'Errico, A.; Trevisani, F.; Wands, J.R. Aspartyl-Asparagyl β Hydroxylase over-Expression in Human Hepatoma Is Linked to Activation of Insulin-like Growth Factor and Notch Signaling Mechanisms. *Hepatology* **2006**, *44*, 446–457. https://doi.org/10.1002/HEP.21272.
119. Zhou, X.; He, Y.; Li, N.; Bai, G.; Pan, X.; Zhang, Z.; Zhang, H.; Li, J.; Yuan, X. DNA Methylation Mediated RSPO2 to Promote Follicular Development in Mammals. *Cell Death Dis.* **2021**, *12*, 653. https://doi.org/10.1038/s41419-021-03941-z.
120. Li, X.; Niu, M.; Su, J.; Ma, Z.; Jin, M.; Qiao, W.; Zhang, Y.; Feng, Y.; An, N.; Hou, Y.; et al. Cloning and Expression Patterns of Neuromedin U and Its Receptors in Pigs. *Neuropeptides* **2017**, *64*, 47–60. https://doi.org/10.1016/J.NPEP.2017.04.003.
121. Choi, K.H.; Park, J.K.; Son, D.; Hwang, J.Y.; Lee, D.K.; Ka, H.; Park, J.; Lee, C.K. Reactivation of Endogenous Genes and Epigenetic Remodeling Are Barriers for Generating Transgene-Free Induced Pluripotent Stem Cells in Pig. *PLoS ONE* **2016**, *11*, https://doi.org/10.1371/JOURNAL.PONE.0158046.
122. Spagnuolo-Weaver, M.; Fuerst, R.; Campbell, S.T.; Meehan, B.M.; McNeilly, F.; Adair, B.; Allan, G. A Fluorimeter-Based RT-PCR Method for the Detection and Quantitation of Porcine Cytokines. *J. Immunol. Methods* **1999**, *230*, 19–27. https://doi.org/10.1016/S0022-1759(99)00114-3.
123. Bogacka, I.; Przała, J.; Siawrys, G.; Kaminski, T.; Smolinska, N. The Expression of Short Form of Leptin Receptor Gene during Early Pregnancy in the Pig Examined by Quantitative Real Time RT-PCR. *J. Physiol. Pharmacol.* **2006**, *57*, 479–489.
