# Supplementary figures and images for: Visfatin Affects the Transcriptome of Porcine Luteal Cells during Early Pregnancy
Source: Int J Mol Sci. 2024 Feb 16;25(4):2339. doi: 10.3390/ijms25042339 (PMC10889815; doi:10.3390/ijms25042339)

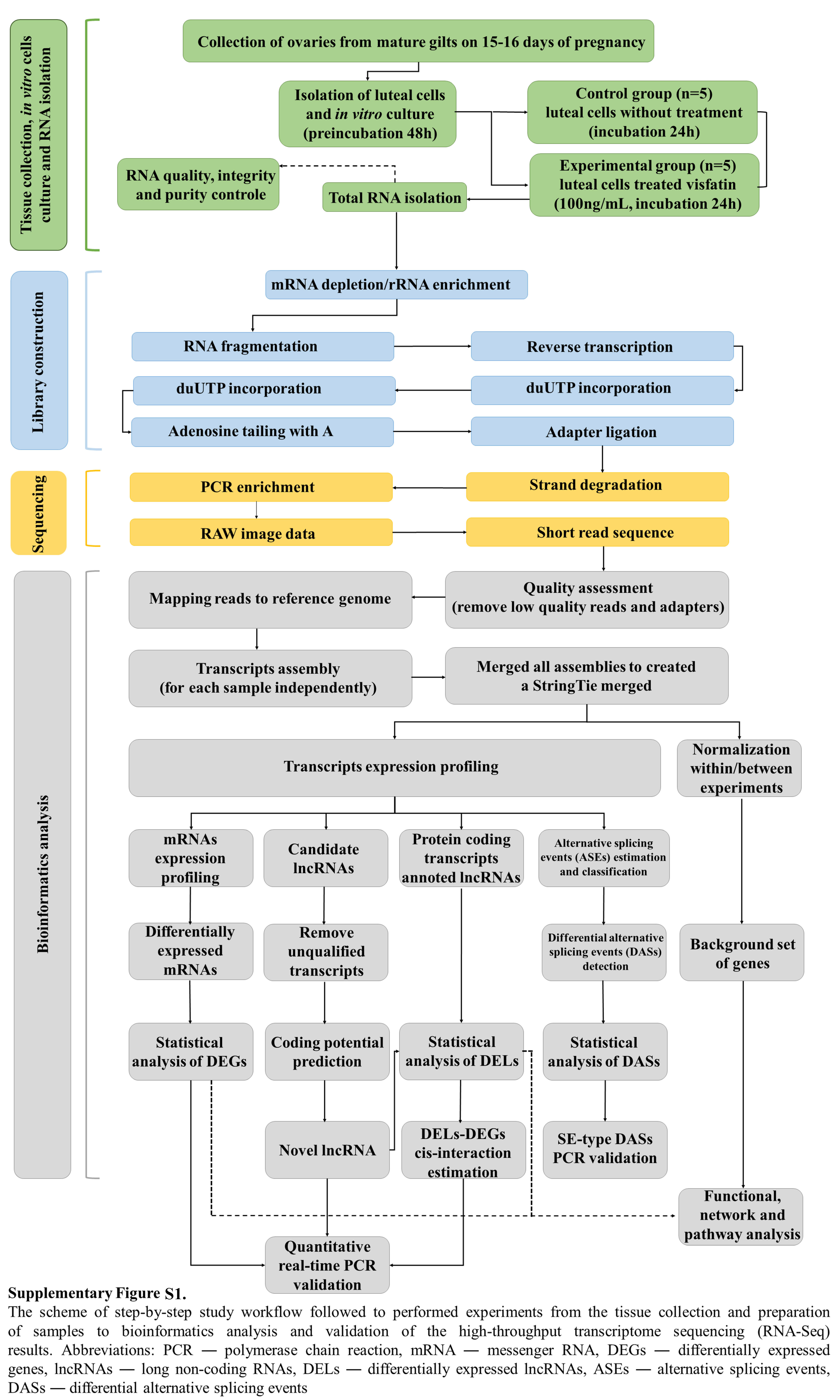

Supplement: Supplementary file 1 [file ijms-25-02339-s001.zip › Supplementary Figure_S1_Pipeline.tif]

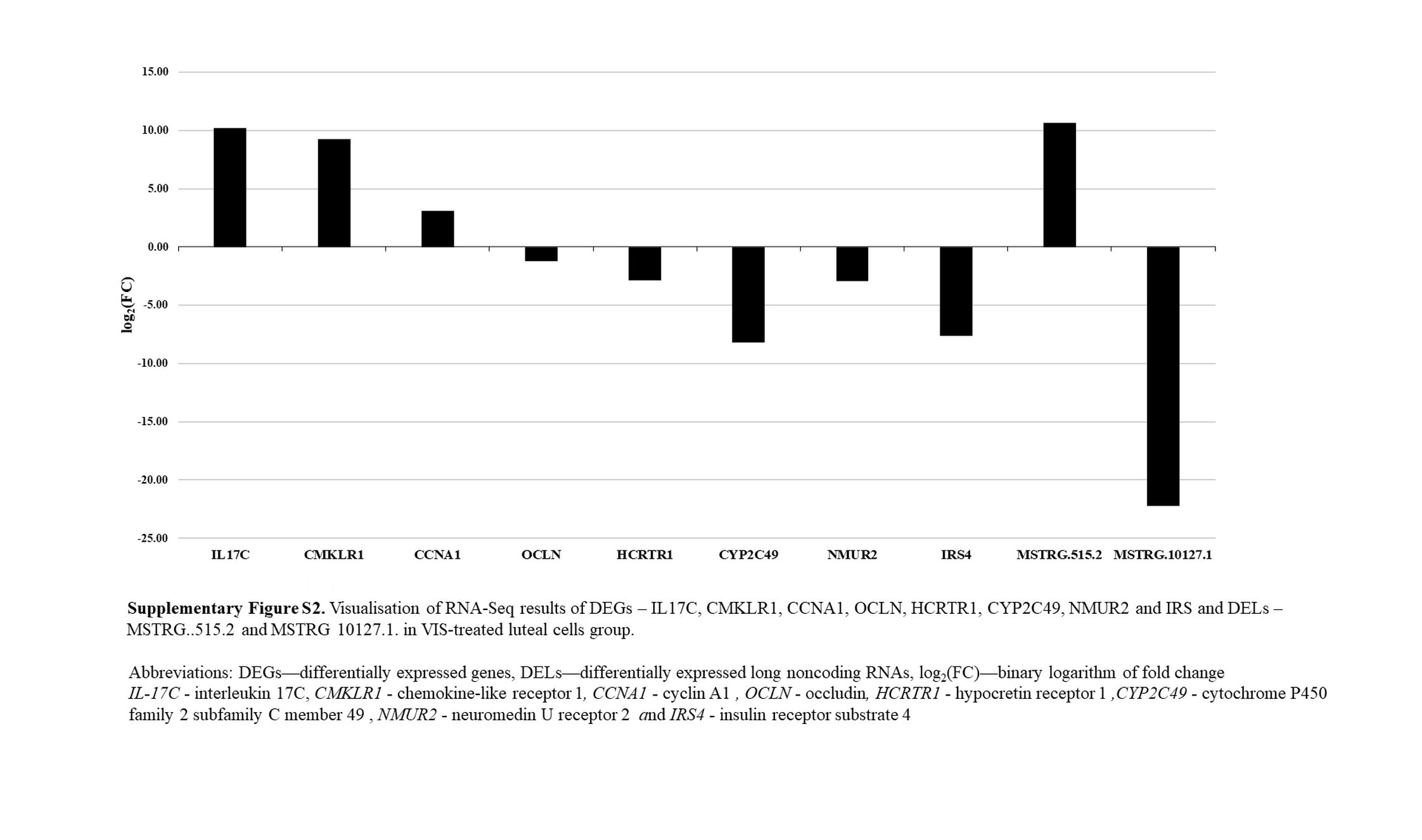

Supplement: Supplementary file 1 [file ijms-25-02339-s001.zip › Supplementary Figure_S2_RNA_Seq.tif]
